# Patient-Derived Organoids as a Model for Cancer Drug Discovery

**DOI:** 10.3390/ijms22073483

**Published:** 2021-03-27

**Authors:** Colin Rae, Francesco Amato, Chiara Braconi

**Affiliations:** 1 Institute of Cancer Sciences, University of Glasgow, Glasgow G61 1QH, UK; colin.rae@glasgow.ac.uk (C.R.); 2496589A@student.gla.ac.uk (F.A.); 2 Beatson West of Scotland Cancer Centre, Glasgow G12 0YN, UK

**Keywords:** organoids, patient, tumour, drug screening

## Abstract

In the search for the ideal model of tumours, the use of three-dimensional in vitro models is advancing rapidly. These are intended to mimic the in vivo properties of the tumours which affect cancer development, progression and drug sensitivity, and take into account cell–cell interactions, adhesion and invasiveness. Importantly, it is hoped that successful recapitulation of the structure and function of the tissue will predict patient response, permitting the development of personalized therapy in a timely manner applicable to the clinic. Furthermore, the use of co-culture systems will allow the role of the tumour microenvironment and tissue–tissue interactions to be taken into account and should lead to more accurate predictions of tumour development and responses to drugs. In this review, the relative merits and limitations of patient-derived organoids will be discussed compared to other in vitro and ex vivo cancer models. We will focus on their use as models for drug testing and personalized therapy and how these may be improved. Developments in technology will also be considered, including the use of microfluidics, 3D bioprinting, cryopreservation and circulating tumour cell-derived organoids. These have the potential to enhance the consistency, accessibility and availability of these models.

## 1. Introduction

Organoids are three-dimensional cultures derived from primary cells which have similar architectures and functions to in vivo organs and may bridge the gap between 2D cultures and in vivo animal models [1]. Organoids retain the histological architectures, genomic landscapes and gene expression profiles of their parental tumours, even after long-term culture, and are amenable for biomarker identification and high-throughput drug screening in vitro [2,3,4]. Tumours are characterized by their heterogeneity and high frequency of genetic and molecular aberrations. Therefore, effective organoid cultures must conserve the characteristics of the original tumour as well as those differing from non-tumour tissue. Importantly, the characteristics of the tumours should also be maintained in the organoids at different passages, and this has been demonstrated in organoids derived from colorectal and gastric cancer [3,5,6].

The relative merits and drawbacks of 2D and 3D cultures have been previously discussed in depth elsewhere [1,7,8,9,10]. While two-dimensional cell line cultures are convenient and easy to use, they are often unrepresentative of primary tumours and do not predict their responses to therapy [11], thus contributing to the low success rate of clinical trials [12]. The use of a relatively small bank of cell lines also does not represent the heterogeneity of tumours, whereas the intratumour and intrapatient molecular heterogeneity of the parental tissue is retained in three-dimensional organoids derived from patient tissue [3,13,14], providing a more valid model and potentially aiding personalized therapy. It is anticipated that these organoids (patient-derived organoids, PDO) will contribute to the approaches of short-term culture and xenotransplantation [7]. Furthermore, organoids derived from patient tissue can be xenografted into immune-deficient mice, and these patient-derived tumour xenografts (PDXs) provide improvements over standard cell-line xenograft models. Although these PDXs are a useful model of metastatic potential, drug responsiveness and tumour architecture [15], their main limitations remain as the cost involved; the time required for tumour growth, which will usually be several months; and they may become clonally distinct from the originating PDOs [11,16]. The growth of tumours in mice is also affected by mouse-specific evolution [17], which influences their therapeutic response, and the limited appropriateness of immunocompromised mice, especially for assessing potential immunotherapies. Although organoids are more costly and require more time to become established when compared to 2D cell cultures or even 3D spheroids, they more closely resemble the primary tumour regarding their morphological, genetic and histological characteristics. One major advantage of using organoids is the potential to obtain tumour and non-tumour tissue from the same patient. Some of the pros and cons associated with the current methods for establishing PDO cultures are summarized in Table 1.

## 2. Establishment of Organoids

Human tumour organoids can be generated either directly from tumour tissues or by the genetic modification of organoids developed from normal tissues, such as using CRISPR gene editing. Organoids from tumour tissues are established by culturing cells, which have been dissociated from the tissue, in 3D semisolid extracellular matrix scaffolds in a defined medium containing appropriate growth factors [18,19]. The presence of a synthetic basement membrane reproduces the in vivo extracellular matrix (ECM) [20]. Growth factor cocktails vary according to the source tumour, but often contain combinations of Wnt, R-Spondin-1 (a Wnt amplifier), epidermal growth factor (EGF), prostaglandin E2, fibroblast growth factor 10 (FGF10), noggin (inhibitor of bone morphogenetic protein signalling), A83-01 (inhibitor of TGF-signalling), SB202190 (p38 inhibitor) and Y-27632 (Rho/ROCK kinase inhibitor) [21]. However, it may be useful in the future to develop media containing the relevant supplements and growth factors, as this has previously been demonstrated to improve the metabolic fidelity and biological relevance for in vitro models [22]. Organoids which maintain the morphological and histological features of the original tumour have been developed to establish and culture PDOs from many common tumour types, including glioblastoma [23], breast [24], pancreas [25], prostate [26,27], liver [28], lung [4,29] and colon [30], with varying degrees of success and reproducibility.

Limitations of organoid cultures include the tissue-specific nature of organoid establishment, with not all tumour tissues being suitable for organoid establishment. The availability of fresh tumour tissue and the expertise associated with their establishment and growth may also preclude some laboratories from utilising these models. The generation of biobanks of organoids may assist these laboratories, but this is often hampered by the lack of standardized protocols for tissue handling, processing and cryopreservation, which may account for the relatively low success rate of the re-establishment of organoids after cryopreservation [31]. However, methods are being developed to overcome this, with the potential of maintaining cancer-stem-cell features that may facilitate organoid development. Refined cryopreservation solutions have also led to improvements in the recovery rates of organoids derived from several tumour types, including pancreatic and breast tumours, retaining their morphological and histological features [24,32]. Some of the limitations of using organoids, which can deter researchers from using these models, or hinder their more widespread use, are summarized in Table 2, along with some potential solutions.

## 3. Drug Screening Using Organoids

Patient-derived organoids can be used to study drug resistance, metabolism, differentiation and cancer gene function, and as will be discussed here, they have been extensively utilized for the screening of potential anti-cancer agents. Drug screening, however, is not limited to cancer therapies, and organoids have also been used successfully to screen drugs for a variety of other diseases, including cystic fibrosis [33] and Zika virus infection [34], further indicating the usefulness of these models. It is anticipated that drug screening using organoids will predict the response in patients, and the value of PDOs in predicting response to chemotherapy, targeted therapy and radiotherapy is being explored. The data obtained in vitro would then be able to predict patient outcomes in the clinic, in contrast to cell lines, which often fail to recapitulate the situation observed in patients. Importantly, it has already been demonstrated that this is often the case, with the response to therapy in patients paralleling the chemosensitivity observed in corresponding PDOs derived from a wide range of cancer types, such as pancreatic, bladder, ovarian, uterine, endometrial, colon, gastrointestinal, head and neck and rectal cancer [5,13,35,36,37,38,39,40,41]. For example, PDOs generated from colorectal cancer patients have been successfully used to evaluate their responses to inhibitors of Tankyrase, which modulate Wnt signalling [42]. Furthermore, novel drugs can be effectively tested in organoids, such as the tubulin-binding drug plocabulin, which was shown to be potently cytotoxic to PDOs derived from colorectal cancer patients [6].

Crucially, the response to drugs also correlated with their known genomic alterations, such as the sensitivity of PDOs from breast and lung cancer patients with BRCA mutations to inhibitors of poly ADP ribose polymerase (PARP), due to their impaired DNA damage response [14,29]. These results in organoids matched with observations in PDX and clinical drug tests. Organoids derived from ovarian cancers harbouring mutations in BRCA1 also predicted their sensitivity to platinum drugs, paclitaxel and PARP inhibitors, in comparison to organoids derived from clear cell ovarian cancer, which were resistant to conventional therapies [43]. Similarly, sensitivity to the epidermal growth factor receptor (EGFR) tyrosine kinase inhibitor drug Erlotinib correlated with EGFR-mutations in lung cancer-derived organoids [29]. Additionally, in PDOs derived from ovarian cancers, the sensitivity to inhibitors of the kinases CHK1 and ATR, which induce replication stress, was associated with the replication fork defects [36]. Interestingly, given the frequency of p53 mutations in cancers, a drug which rescues p53 function (ReACp53) induced apoptosis in organoids with mutations in p53 [44]. Furthermore, the response of organoids to radiation in these ex vivo models also correlated with the response observed in matched patients [40,41], and can be used to test the feasibility of new radiotherapeutic approaches [9].

Importantly, unwanted side-effects in normal tissues can also be studied in organoid models [45]. These adverse drug reactions are a major clinical problem, limiting the dose and effectiveness of drugs. Organoids have been used to accurately predict potential toxicities to many of the non-target tissues affected, including intestinal, renal, hepatic, cardiac and neuronal models [46]. A method for establishing organoids from non-tumour tissue was also recently described [47], demonstrating that it is possible to culture non-tumour PDOs, and this will provide an important tool for evaluating the effects of therapy on tumour and non-tumour tissues from the same patient. For example, the induction of hyposalivation, a serious side effect of radiotherapy for head and neck cancer, can be predicted from organoids derived from patients’ salivary glands [48]. Similarly, 3D cultures of organoids derived from liver have been used to evaluate drug-induced hepatotoxicity [28], organoids from kidney predicted nephrotoxicity in response to cisplatin [49] and drug-induced neurotoxicity was investigated in brain organoids [50]. Potential side effects of therapy can also be tested using matched organoids from tumour and normal tissue. This was demonstrated by Driehuis et al., who used PDOs from head and neck tumour tissue and adjoining non-tumour tissue to evaluate the effects of EGFR-targeted photodynamic therapy [51]. This study showed that EGFR expression in tumour tissue mimicked that in patients and correlated with the response to therapy.

## 4. Cancer Biomarkers

Cancer biomarkers are an important tool for stratifying patients and predicting their response to therapy. Although biomarkers have been successfully identified for some cancer types, such as breast cancer, which has led to the optimization of drug therapy, many other cancer types have no or few biomarkers which predict response to therapy. Liquid biopsies, particularly blood samples, are non-invasive and may be utilized to detect potential biomarkers, such as circulating tumour cells, methylated DNA, cell free DNA, circulating RNA (including microRNA) and extracellular vesicles (including exosomes) [52,53]. The identification of potential predictive biomarkers will help to understand the sensitivity or resistance to therapies, mechanisms of drug resistance and interactions of combination therapies, and this will be augmented by the use of organoid models, as has been demonstrated in several cancer types.

Crucially, the expression of characteristic biomarkers was more likely to be retained in PDOs than two-dimensional culture models [54]. For example, the response of colon cancer organoids to the inhibition of the histone methyltransferase EZH2 was correlated to expression of the pro-apoptotic *BIK* gene [55]. Similarly, the resistance of cholangiocarcinoma organoids to inhibitors of the heat shock protein HSP90 appeared to be mediated by the expression of the microRNA miRNA21 [2]. Levels of DNA methyltransferases were identified as a biomarker of sensitivity to decitabine using breast cancer organoids, indicating its mechanism of action and guiding drug selection in the clinic [56]. Gene expression profiling of organoids derived from biliary tract carcinomas has also identified several novel prognostic biomarkers, predicting outcome and drug sensitivity in these cancers [57]. Importantly, in the absence of suitable biomarkers, the effects of chemotherapy and the development of resistance could be investigated using organoids derived from patients at different stages of their therapy. Furthermore, the use of organoid models has been used to predict interactions of combination therapies. Although the use of EGFR inhibitors has been unsuccessful in unselected gastro-oesophageal adenocarcinoma patients, the EGFR copy number can be measured using tissue and liquid biopsies, allowing the selection of patients based on EGFR status. The interaction of drugs targeting EGFR with chemotherapy has recently been demonstrated using PDOs from these patients, with an antagonistic effect observed between anti-EGFR agents and the anthracycline chemotherapeutic epirubicin [58]. This will have important implications for the design of future combinatorial trials and indicates that patient selection using biomarkers can be effectively modelled in patient-derived organoids. The identification of diagnostic biomarkers may also be possible using organoids, as demonstrated by the detection of mutations in cell-free DNA (cfDNA) from conditioned media from organoids derived from pancreatic ductal adenocarcinoma [59]. Importantly, these genetic alterations recapitulated the mutational profile of the primary tumour and indicate the potential use of cfDNA as a diagnostic marker.

## 5. Tumour Microenvironment

Although organoids can replicate the morphology of source tissues, one common limitation is that they are often not cultured in conditions which replicate the tumour microenvironment (TME). The TME is comprised of the extracellular matrix (ECM) and non-tumour cells and is highly variable depending on tumour type and location. ECM molecules include matrix proteins, glycoproteins, glycosaminoglycans, proteoglycans, growth factors and other secreted proteins, such as cytokines and metabolites [12]. The composition of this matrix can influence the cell’s sensitivity or resistance to drugs or radiation due to alterations in the levels of hypoxia, pH, the presence of inflammatory cells or cell-adhesion-based drug resistance [60,61].

Tumour-associated stromal cells are key contributors to the TME, and there is substantial crosstalk between these cells. These cells include mesenchymal cells, fibroblasts, endothelial cells, adipocytes and immune cells, which secrete a variety of soluble factors, such as pro-inflammatory cytokines, matrix metalloproteinases and growth factors [62]. It has been shown that the presence of stromal fibroblasts in co-cultures with organoids enhanced the maintenance of characteristics of 3D models of human tongue cancer, which were more comparable to native tissues than co-cultures where these cells were not present [63]. Therefore, co-culture systems which accurately replicate the in vivo situation are required in order to determine how the TME affects the response to potential therapeutic agents. Co-cultures of PDOs with these cells will allow researchers to investigate the interaction of tumour cells and their surrounding cells, and it has been shown that the co-culture of PDOs with cancer-associated fibroblasts (CAFs) affects their response to chemotherapy and immunotherapy [64,65]. Similarly, the co-culture of PDOs with lymphocytes permits researchers to study their interactions with tumour cells. Interestingly, this approach has also been utilized to generate patient-specific tumour-reactive T cells from peripheral blood lymphocytes [66], allowing the interrogation of tumour sensitivity to immunotherapy. Indeed, the use of PDOs in co-cultures is likely to facilitate in vitro screening of cellular immunotherapies, allowing the real-time determination of patient sensitivity to these treatments [67]. Co-cultures of PDOs derived from circulating tumour cells with CAFs also resulted in the greater expansion of these cells in vitro [68]. Recently, a method has been developed where organoids derived from a variety of cancers were grown at the air–liquid interface, allowing PDOs to retain CAFs and lymphocytes with similar features and activity to those in the tumour of origin [69]. This method closely replicates the tumour microenvironment and preserves the immune profile required for modelling personalized immunotherapy.

One mechanism whereby the CAFs affect resistance to therapy is the dysregulated expression of microRNAs (miRNAs), small non-coding RNAs, which can regulate the expression of many target genes. The upregulated expression of miRNA21 in CAFs from pancreatic ductal adenocarcinoma (PDAC) patient tumours has been reported, and this was associated with poor prognosis [70]. Similarly, the upregulation of miRNA21 and miRNA221 in CAFs appeared to confer aggressiveness to pancreatic cancer cells [71], and was associated with the hyperactive signalling of the mitogen-activated protein kinase (MAPK) pathway in breast cancer cells and decreased response to therapy [72]. Conversely, the downregulation of miRNA15 and miRNA16 in CAFs promoted the growth and progression of prostate cancer [73]. The involvement of miRNAs in CAF activity was further demonstrated by the transformation of normal fibroblasts to CAF-like cells by miRNAs [74,75]. Furthermore, it has been demonstrated that modulating the expression of miRNAs can mediate sensitivity to anti-cancer therapy in organoid models [2].

An alternative ex vivo system, similar to organoid culture and incorporating the tumour microenvironment, is the use of precision-cut liver slices (PCLS). These can be obtained from both animals and humans and have been used to assess drug toxicity and metabolism. These models retain the three-dimensional architecture and mRNA expression of whole liver and have the advantage that hepatocytes and non-parenchymal cells are cultured simultaneously, thus providing a more accurate model of the TME than many other cell culture models, with cell–cell and cell–extracellular matrix interactions being preserved. When tissue slices from hepatocellular cancer are used, they can be utilized for preclinical drug testing and offer the benefit of being able to investigate cancer–immune cell interactions by co-culturing with matched immune cells [76]. The use of PCLS can also be used to identify cell signalling pathways involved in metastasis and invasion in the liver [77]. In contrast to organoids, however, these models are limited by their short-term survival in culture of only around 7 days, with loss of cell viability observed within 1 day of culture and necrosis occurring from day 2 after culture [78]. However, it has been suggested that the gene expression profiles of these slices, and their response to exogenous stimuli, could be maintained in ex vivo culture for up to 15 days [79], demonstrating that they may have potential applications for drug screening.

## 6. Emerging Technologies

### 6.1. High-Throughput Assays

**Figure 1:**
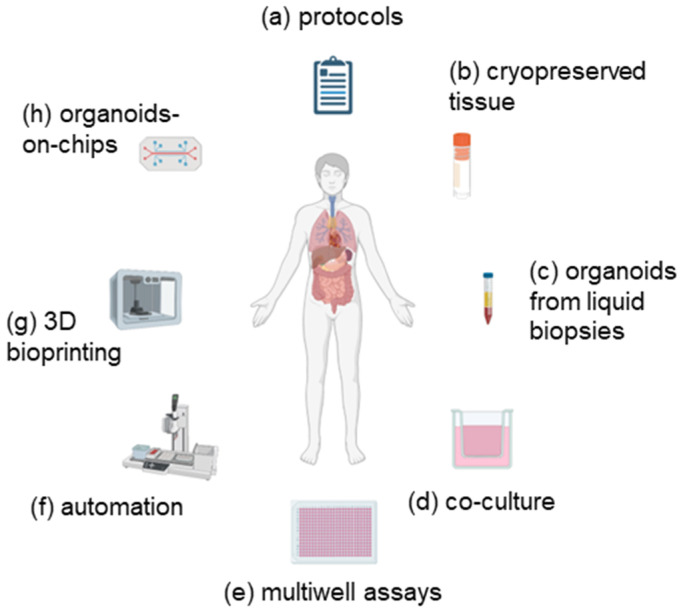
Use of emerging technologies to improve organoid assays. In order to overcome the limitations of organoid cultures and improve their efficiency, new developments are required. These are described in more detail in the text and may include (**a**) the use of enhanced or standardized protocols; (**b**) use of cryopreserved tissue; (**c**) establishment of organoids from circulating tumour cells; (**d**) co-culture with components of the tumour microenvironment; (**e**) assays in multi-well formats; (**f**) automated processes for establishment and testing; (**g**) 3D bioprinting; (**h**) use of organoids-on-chips. Image created with Biorender.com.

The emergence of technologies which have the potential to overcome the limitations of organoid culture and provide organoids as 3D models in automated, large-scale cultures without the cost, time and expertise associated with primary cells is summarized in Figure 1. The use of high-throughput screening assays is essential to develop new drugs and combinations of therapies, and, therefore, the potential application of PDOs in this area is currently being evaluated. These may include 3D bioprinting and microfluidic systems, co-culture and liquid handling robots [80] among other innovations. A protocol for the drug screening of organoids derived from human oesophageal and colorectal cancers in 384-well plates has been described [81]. Yan et al. assessed 37 anticancer drugs in 9 gastric cancer organoids and demonstrated that the screening of organoids was feasible [3]. Therefore, it appears to be feasible to employ organoids in these assays, and it is hoped that the modification of the culture conditions will allow the automation of the process, thus reducing the variability while further maximizing the assays performed on each sample. An automated platform for the high-throughput screening of patient-derived 3D organoids has recently been described; however, this system was not able to use organoids embedded in the extracellular matrix [82]. Using an automated platform, organoids derived from single cells from colon cancer tissue were established in a 384-well plate format which could be used for testing drugs in a high-throughput assay [30]. This method required the culture of the organoids in 12-well plates for expansion before dissociation and re-plating single cells suspended in Matrigel in 384-well plates. Similarly, Pauli et al. assessed organoid viability in a high-throughput screen, which was then validated in patient-derived xenograft models [35]. Many preclinical experimental models rely on the measurement of only cell viability, which is often insufficient to determine the effectiveness of potential chemotherapeutics. Therefore, the development of high-throughput assays which measure both cell death and cell growth rate in response to drugs is likely to improve the success rate for oncology drugs. Importantly, it has been demonstrated that these assays can be applied to more complex, translationally relevant models, such as organoids [83]. A further development is the culture of organoids in “mini-rings”, where the wells are seeded in Matrigel around the rim of the wells [84]. This method has allowed the viability, number and size of organoids derived from ovarian cancers to be measured after treatment with kinase inhibitors in a 96-well plate format and offers the advantage of requiring only small cell numbers, reducing the time for expansion and maximising the assays performed on each sample. The generation of these organoids in mini-rings facilitates media exchange and drug treatment, thus permitting histopathological characterization, and should facilitate automated sample handling, thus increasing their usefulness in high-throughput screening assays [85]. Crucially, the drug sensitivities observed using this method appeared to correlate to patient responses [84]. It is likely that similar innovations will permit the fully automated screening of organoids using small cell numbers, in multi-well formats, and in short (1–2 week) timescales required for the clinic.

### 6.2. Organoids-on-a-Chip

Microfluidic devices have been developed which closely mimic the tissue–tissue interfaces, chemical gradients and vascular perfusion of the body. These devices, which allow the supply of oxygen and nutrients, while removing waste products, provide a more accurate recapitulation of organ functionality compared to conventional two- or three-dimensional culture systems [86]. The use of a microengineering approach has led to the development of so-called “organ-on-a-chip” platforms, which contain hollow microchannels populated by living cells from one type of tissue or combinations of two or more tissue types. These microfabricated cell culture devices have been created to model normal human organs which retain many of the key anatomical and functional features, and have been shown to more closely resemble the in vivo organ than the organoids from which they were derived [87,88]. Organs-on-a-chip provide an accurate replication of the cell–cell interactions of the native tissue and can closely mimic the properties of the tissue of origin, such as cell adhesion, migration, differentiation and function [89], and it is believed that their development has the potential to increase the success rate and speed of drug development [90,91].

Similar technology may offer a solution to the variability observed in organoid culture, in terms of size, structural organization, functional capacity and gene expression [88]. Therefore, it is hoped that the combination of organ-on-a-chip technology with developments in organoid culture will substantially benefit these in vitro models. A variety of organ-specific disease states can be effectively simulated using microfluidic organoid chips [86], as well as normal development and differentiation, such as that demonstrated in human brain organoids [92]. Using organoids derived from tumour tissue, it is believed that a similar approach can be used to model tumours. An organoid-on-a-chip has also been developed which was able to deliver drugs to organoids derived from breast cancer tissue through a perfusable blood vessel network [93]. This system allowed the assessment of features of tumour progression, including migration and proliferation. Another microfluidic platform with vascularized microtumours was used to distinguish between drugs which were effective in disrupting vascular networks [94]. Similar platforms, which reflect the genetic characteristics of each patient, will provide a useful tool for the rapid assessment of drug sensitivity in a clinically relevant timeframe, leading to the development of personalized medicine. The use of fully automated microfluidics systems will also provide a platform which can be utilized in drug testing applications. This has previously been demonstrated using brain and liver organoids [92,95], and apoptosis induction has been measured in organoids from small-cell lung tumours in response to the chemotherapeutic agents cisplatin and etoposide [96].

The interaction between the tumour and its microenvironment, which affects the response to drugs, can also be modelled using a microfluidic system. These systems have been used to provide an insight into the interaction between circulating tumour cells and the vasculature by demonstrating the promotion of adherence of these cells to endothelium by chemokine CXCL12 [97]. The interaction of cancer cells with components of the tissue microenvironment was also effectively evaluated in a study by Montanez-Sauri et al., where breast carcinoma cells were co-cultured with fibroblasts and combinations of different ECM proteins [98]. By growing organoids in chips lined by normal non-tumour cells, these devices can be used to model tumour progression, expansion, invasion and metastasis [86,99]. For example, Ying et al. demonstrated that hepatocyte growth factor from cancer-associated fibroblasts affected cell signalling in lung cells, promoting chemoresistance to paclitaxel [100]. Similarly, using tissue from lung cancer patients cultured in 3D gels within a microfluidic device and co-cultured with stromal cells, sensitivities to anticancer drugs were investigated [101]. This study also allowed the optimization of doses and combinations of drugs for patients. It is, therefore, becoming apparent that microfluidic organoid chip technologies, which create tissue–tissue interfaces and replicate the tumour microenvironment, represent an effective model for the evaluation of anticancer therapies and development of personalized treatments, and have the potential to better predict the clinical effectiveness of drugs than animal models.

### 6.3. Bioprinting

High-throughput screening will be also enhanced by the development of techniques which maintain the intact morphology of organoids for histological processing. One recent study demonstrates the use of a hydrogel-based transfer device for the histological processing and immunostaining of arrays of cerebral organoids, which retained the organoid’s features, thus improving efficiency and increasing throughput [102]. Alternatively, 3D bioprinting, the layer-by-layer deposition of bioinks, such as tissue spheroids, ECM components and cell-laden hydrogels [103], generates 3D constructs which can be used as a drug screening tool. Hydrogels offer several advantages for bioprinting, as they are durable, allow adhesion of cells and can change from liquid to semi-solid [104]. Reid et al. demonstrated the adaption of 3D bioprinting to culture organoids of mammary epithelial structures printed into hydrogels [105]. This generated consistent and repeatable structures using technology which could be replicated in other laboratories. Similar 3D bioprinted cancer models have since been generated for glioblastoma, pancreatic adenocarcinoma, cervical tumours and hepatocarcinoma [103]. Using magnetic drives to aggregate cells into spheroids or organoids, 3D bioprinting may also allow the production of these 3D models to be scaled up into multi-well plates for 96-, 384- and even 1536-well formats [106], thus supporting fully automated, high-throughput screening. A further development in bioprinting was reported by Chadwick et al., who generated a 4D printed array for organoids derived from glioblastoma patients which could be used to assess drug sensitivity [107]. In this case, 4D refers to 3D printing with materials which can respond to stimuli, such as heat, by altering their 3D shape. These printed arrays used expandable/collapsible smart material to test combination therapies, including drug targeting mTOR, PI3K, PTEN and DNA damage response in PDOs derived from glioblastoma, and eliminated the manual transfer of 3D arrays and increased the compatibility with high-throughput assays [107].

### 6.4. Circulating Tumour Cells

Another expanding area of research is the generation of PDOs from alternative sources, such as from cells available through liquid biopsies, due to their relative ease of attainment [52]. Liquid biopsies provide a source of circulating tumour cells (CTCs), which have metastasized from the primary tumour into the patient’s blood. These biopsies are inexpensive and permit repeated sampling, unlike traditional sources of biopsies, thus allowing the possibility of modelling disease progression and pre- and post-treatment effects in the same patient. Although CTCs are rare (approximately 1 in 1,000,000 circulating cells), they are useful in the diagnosis and prognosis of various cancers, and several methods have been developed to isolate these cells for the generation of organoids. These methods rely on the biological and physical characteristics of CTCs and often include the use of immunoaffinity-based enrichment techniques utilising the expression of common CTC markers, or density gradient centrifugation and filtration, which rely on the density and size of CTCs compared to other blood components [21].

The growth of organoids derived from CTCs, which retain the histological and genetic characteristics of the primary tumours, has been demonstrated from liquid biopsies obtained from patients with several cancer types [26,27,68]. These studies indicate that the use of CTCs has high potential for drug screening and personalized medicine and will allow multiple organoid lines to be established from the same patients over time or during the course of therapy. For example, 3D cultures of CTCs derived from prostate and lung cancer patients have been shown to be applicable for the screening of drug sensitivity, and can predict treatment response in vitro [26,108]. Furthermore, it has been demonstrated that CTCs from small cell lung carcinoma patients, grown as explants in mice, mirror the donor patient’s sensitivity or resistance to chemotherapy [109]. By combining technologies, Khoo et al. described a method for the expansion and culturing of clusters and spheroids of CTCs using a microfluidic device [110]. This method created a tumour microenvironment that comprised feeder blood cells from the same patient blood sample in a microfabricated device, leading to a culture efficiency of greater than 50%, and providing a platform for drug screening. Furthermore, the phenotype of the cluster correlated with patient response to treatment and was established after just 2 weeks of cluster formation, allowing the potential for personalized therapy and serial sampling to monitor disease [110].

### 6.5. Cryopreservation

Collaborations between laboratories will be improved by methods which allow the shipment of materials between them, such as cryopreserved tissues or organoids. In order to improve the recovery rate from the cryopreservation of patient-derived organoids, several studies have aimed to develop protocols which increase this rate. Methods have recently described the use of commercially available freezing media which may increase the possibility of using tissue frozen before deriving organoids. This has been demonstrated using tissue from pancreas, colon, thyroid, lung, renal and liver tumours, where tissue was frozen in cryopreservation solution and showed that organoid establishment was possible from the thawed tissue [32,111]. It has also been demonstrated that organoids established from both fresh and frozen endometrial tissue had comparable organoid formation, morphology, expression of markers, proliferation rates and response to treatment [112]. This would increase the accessibility of tissue for organoid culture and has the potential to allow many more researchers to participate in studies using these tissues. Similarly, in an attempt to establish organoids from breast cancer tissue from patients in remote or poorly resourced regions, Okoli et al. demonstrated that cryopreservation after the processing of the tissue had no detrimental effect on tissue viability [113]. A further development in this area is the use of “in-plate cryopreservation”, which can be applied to organoids. Prinelli et al. recently described a method for the cryopreservation of human hepatic and colon organoids in the basement membrane matrix in 24-well plates [114]. Cell viability, physiology and drug sensitivity in these organoids were maintained after thawing.

## 7. Conclusions

Although patient-derived organoids represent an imperfect model, they are an important area of research, as they recapitulate many functional and genetic characteristics of the tissues from which they were derived. They have the advantage of being able to be passaged, frozen, resuscitated and preserved and may be used in high-throughput screening. PDOs are useful tools for assessing mutational profiles, and we believe one of their main applications will be the potential of the PDO as a model for testing drugs and, in particular, personalized therapy. These organoid models offer a cheaper and more rapid alternative to xenograft models and are more realistic in vitro models than traditional two-dimensional cell culture models. However, there are several limitations to these models, some of which can be overcome through the development of standardized protocols and reagents. It is hoped that, in the future, developing technologies, such as organoids-on-chips, 3-D bioprinting, improved handling and imaging techniques, and the establishment of PDOs from small biological samples, such as liquid biopsies, will continue to improve the reliability and utilization of these models.

## Figures and Tables

**Table 1:** Advantages and disadvantages of the more common methods of establishing patient-derived organoids.

Advantage	Disadvantage
Cheaper and more rapid than xenografts	More expensive than 2D culture
Retain key features of tumours	Time-consuming
Allows repeated sampling from patients	High-throughput screening not fully developed
Can predict response to therapy	Limited availability of expertise

**Table 2:** Limitations of organoid establishment and potential solutions. The widespread use of organoids may be hindered in some laboratories by lack of expertise, protocols and availability of suitable tissue, and some simple solutions may be possible to overcome these.

Limitations	Potential Solutions
Labour intensive	Development of high-throughput systems
Non-standardized methods	Adaption of standard protocols
Different culture medium between labs	Formulation of commercial media/supplements
Collaboration between distant labs	Improved freezing protocols
Absence of tumour microenvironment	Evolution of co-culture models
Availability of tissue	Increased access to biobanks Use of circulating tumour cells

## Data Availability

Please refer to suggested Data Availability Statements in section “MDPI Research Data Policies” at https://www.mdpi.com/ethics.
